# Long‐term adherence and response to botulinum toxin in different indications

**DOI:** 10.1002/acn3.51225

**Published:** 2020-12-01

**Authors:** John‐Ih Lee, Alexander Jansen, Sara Samadzadeh, Ulrike Kahlen, Marek Moll, Marius Ringelstein, Giulia Soncin, Hans Bigalke, Orhan Aktas, Alexia‐Sabine Moldovan, Julia Waskoenig, Sebastian Jander, Michael Gliem, Alfons Schnitzler, Hans‐Peter Hartung, Harald Hefter, Philipp Albrecht

**Affiliations:** ^1^ Department of Neurology Medical Faculty Heinrich Heine University Düsseldorf Moorenstrasse 5 Düsseldorf D‐40225 Germany; ^2^ Department of Neurology Center for Neurology and Neuropsychiatry LVR‐Klinikum Heinrich Heine University Düsseldorf Düsseldorf Germany; ^3^ Toxogen GmbH Hannover Germany; ^4^ Institute of Clinical Neuroscience and Medical Psychology Heinrich Heine University Düsseldorf Germany

## Abstract

**Objective:**

The objective of the study was the analysis of adherence and self‐perceived treatment response to long‐term botulinum neurotoxin type A (BoNT‐A) treatment in different neurological indications.

**Methods:**

In this retrospective, monocentric, observational study, cross‐sectional and longitudinal data of 1351 patients documenting 20705 injection appointments at the BoNT outpatient clinic of Heinrich Heine University Duesseldorf between 1989 and 2014 were retrospectively analyzed. Patients had been treated with BoNT for neurological conditions, including cervical dystonia (CD), blepharospasm (BSP), other dystonia (ODT), hemifacial spasm (HFS), and spasticity (SPAS). The parameters longitudinally analyzed for the entire cohort were therapy duration as well as the mean and cumulative BoNT‐A dose. Cross‐sectionally, for subgroups of at least 721, patients’ global self‐perceived quality of health and life, global self‐perceived reduction of symptoms by BoNT‐A treatment as well as the clinical global impression were evaluated. Furthermore, mouse hemidiaphragm assay antibodies (MHDA‐ABs) were analyzed in a subgroup.

**Results:**

The mean treatment duration was 4.58 years (95% CI 4.32–4.84), and 678 (50.2%) therapy dropouts of 1351 patients occurred within the first 8 years. Therapy adherence and self‐perceived symptom reduction in long‐term BoNT‐A treatment over the years were significantly longer in BSP, HFS, and CD patients than in ODT and SPAS patients.

**Interpretation:**

The treatment indication determines long‐term adherence and self‐perceived symptom reduction in BoNT‐A therapy, which are better in BSP, HFS, and CD patients than in ODT and SPAS patients. MHDA‐ABs had a significant impact on global self‐perceived symptom reduction, but with only a limited degree.

## Introduction

Botulinum neurotoxin type A (BoNT‐A) formulations, such as ona‐BoNT‐A (Botox^®^), abo‐BoNT‐A (Dysport^®^), and inco‐BoNT‐A (Xeomin^®^), are widely used in the long‐term therapy of different neurological disorders, including, but not limited to, cervical dystonia (CD), dystonia other than cervical dystonia (ODT), blepharospasm (BSP), hemifacial spasm (HFS), and spasticity (SPAS).

In all of these conditions, increased muscle tone leads to abnormal movement and potentially pain, causing both physical and/or emotional disability, with often devastating effects on the patients’ quality of life (QoL).^1^ Over the past few decades, BoNT‐A therapy has been established as one of the most important therapeutic options for these disorders.

Although the efficacy and safety of BoNT‐A injections have been evaluated in numerous randomized, placebo‐controlled trials,^2^, ^3^, ^4^, ^5^, ^6^ real‐world evidence regarding patient‐reported outcomes (PROs) and long‐term adherence to therapy is scarce.^1^, ^7^, ^8^, ^9^ PROs obtained in the clinical routine are increasingly considered, as they reflect patients’ self‐perceived QoL and efficacy of therapy. In addition, the clinical readouts applied in controlled trials do not always reflect patients’ interests, needs, and concerns in a real‐world setting.^1^, ^10^

Therefore, adherence and self‐perceived treatment success as reported by patients in long‐term BoNT‐A treatment for different neurological indications have attracted great interest.

## Methods

### Standard protocol approval, registration, and patient consent

This study was approved by the local ethics committee of the Heinrich Heine University Duesseldorf, Germany (#5820R and #4085R). In accordance with the Declaration of Helsinki, written informed consent was obtained from all patients on regular follow‐up at the outpatient clinic, and oral informed consent was obtained in a standardized manner prior to all telephone interviews that were performed with patients no longer on follow‐up.

### Patients

Data from 1351 patients documented and treated at the BoNT outpatient clinic of the Department of Neurology, University Hospital at Heinrich Heine University Duesseldorf, Germany between 1989 and 2014 were retrospectively and cross‐sectionally analyzed. Only patients with neurological indications and at least one BoNT‐A injection were included. The mean age of all patients at treatment beginning was 55.40 ± 14.54 SD years.

Patients were divided into five subgroups: CD (*n* = 527 with a mean age at treatment beginning of 53.44 ± 13.41 SD years), BSP (*n* = 173 with a mean age at treatment beginning of 62.53 ± 12.42 SD years), ODT (*n* = 283 with a mean age at treatment beginning of 51.19 ± 15.46 SD years), HFS (*n* = 184 with a mean age at treatment beginning of 61.01 ± 13.48 SD years), and SPAS (*n* = 184 with a mean age at treatment beginning of 55.03 ± 15.16 SD years). The ODT subgroup included patients with Meige syndrome, oromandibular and oropharyngeal dystonia, focal or segmental dystonia of the extremities, and generalized dystonia.

### Assessments and readouts

All assessments were conducted by the treating physician at the scheduled reinjection appointments in our BoNT outpatient clinic.

The European Organisation for Research and Treatment of Cancer (EORTC) QLQ‐C30 (version 3.0) questionnaire was designed and validated to assess health‐related QoL in the previous week.^11^ However, as it was not feasible to perform an entire questionnaire due to time constraints, only items 29 and 30 were assessed, inquiring about the global self‐perceived general quality of health and life, respectively [using a scale ranging from (1) “very poor” to (7) “excellent”].

Further items assessed were the self‐perceived reduction of symptoms from the patient’s point of view over the last BoNT injection interval and the clinical global impression regarding the patient’s symptoms from the treating physician’s point of view at the appointment for BoNT reinjection. These items were rated on a scale ranging from 0% (no symptom reduction) to 100% (complete symptom reduction) compared to the status prior to the first BoNT‐A therapy (from the patient’s point of view) and compared to the maximum possible manifestation of the treated disorder (from the physician’s point of view).

We used our local database to analyze longitudinal data of 1351 patients regarding therapy duration, the number of injections, and injected mean unified dose units (uDUs). Furthermore, we cross‐sectionally investigated the global self‐perceived quality of health in 722 patients, global self‐perceived QoL in 721 patients, global self‐perceived reduction of symptoms by BoNT‐A treatment in 734 patients, and clinical global impression regarding symptoms evaluated by the treating physician in 733 patients.

BoNT‐A antibody testing was performed by an independent blinded contractor (BSL Bioservice Scientific Laboratories, Planegg, Germany) using two consecutive sandwich enzyme‐linked immunosorbent assays (ELISAs) for screening and confirmation of binding antibodies, as previously described.^12^ Samples identified as ELISA‐positive were then transferred to another contractor (Toxogen GmbH, Hannover, Germany) for discrimination of binding antibodies and the mouse hemidiaphragm assay antibodies (MHDA‐ABs).^13^

In a previous analysis, we reported the prevalence of MHDA‐ABs against BoNT‐A in 596 long‐term treated patients, but we did not analyze adherence to therapy or patient satisfaction.^14^ Therefore, in the study presented here, we included information on the MHDA‐AB status of these patients in our analyses to investigate the effect of MHDA‐ABs on the outcomes of this study.

In line with previous studies,^14^, ^15^, ^16^ unified dose units were calculated by multiplying the injected inco‐BoNT and ona‐BoNT units by 2.5 to reach comparability with abo‐BoNT.

### Analysis and statistics

SPSS statistics package (IBM) 20 was used for statistical analysis. In addition to descriptive statistics, Kaplan–Meier analysis of the five disorder subgroups was performed using Cox proportional hazard models to compare the trajectories of the different groups. To compare group characteristics, analysis of covariance (ANCOVA) with Bonferroni confidence interval adjustment correcting for age and treatment duration was performed. For nonparametric testing, the Wilcoxon or Mann–Whitney *U* test was used with Bonferroni correction for multiple group comparisons, as indicated in the Results section. Spearman correlation analysis was performed to analyze the association between the different parameters. A stepwise multilevel linear regression model was used to detect factors significantly influencing the BoNT dose. For all analyses, *P*‐values < 0.05 were considered significant. Power analyses to estimate the sample sizes necessary to obtain an actual power (1‐beta) of 0.95 (two‐tailed) were performed with G*Power (Version 3.1.9.2) to determine possible reasons for negative results in subgroup analyses when applicable.

## Results

We analyzed the data of 1351 patients with altogether 20705 injection appointments who received at least one BoNT injection at our center. These injection appointments were distributed as follows: 9092 CD, 3304 BSP, 2832 ODT, 3274 HFS, and 2203 SPAS treatments. The maximum duration of treatment was 23 years, with a mean of 4.58 (95% CI 4.32–4.84) treatment years.

### Long‐term adherence to therapy by treatment subgroups

**Figure 1:**
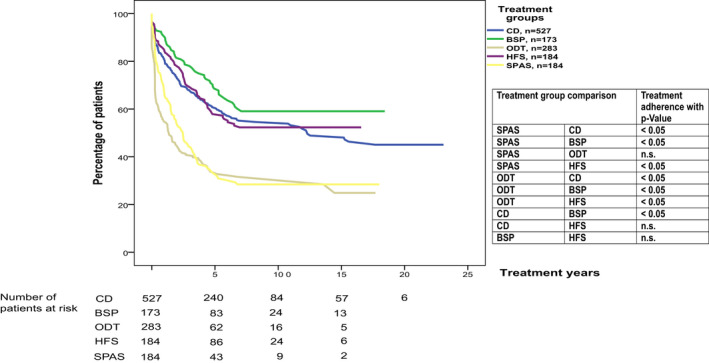
Kaplan–Meier curves show the probability of remaining on treatment for the different indications (CD, BSP, ODT, HFS, and SPAS). Therapy adherence comparisons of the indication groups over the years are presented, including *P*‐values calculated by Cox proportional hazard models correcting for age at the first injection. Number of censored patients: CD 302, BSP 115, ODT 106; HFS 107, SPAS 67.

Kaplan–Meier analysis of treatment duration for the different disorders showed that the majority of dropouts occurred within the first 8 years of therapy for all indications. Dropouts were defined as patients who had not returned for reinjection for ≥6 months since the last BoNT injection. We observed significantly higher rates of treatment dropouts in ODT and SPAS than in the three other indications (*P* < 0.05, Cox proportional hazard models correcting for age at first injection). Fifty‐nine percent of the 173 BSP, 52% of the 184 HFS, 46% of the 527 CD, 25% of the 283 ODT, and 28% of the 184 SPAS patients remained on treatment for over 16 years. The longest follow‐up data of 23 years were available for CD patients, with 45% treatment adherence (Fig. 1).

### Global self‐perceived symptom reduction

To investigate the dynamics of treatment success from the patients’ point of view, we performed a Kaplan–Meier analysis of the probability of patients remaining among the group declaring ≥50% global self‐perceived symptom reduction compared to that in the pretreatment situation over the years of treatment. The analysis was performed independently by treatment groups.

**Figure 2:**
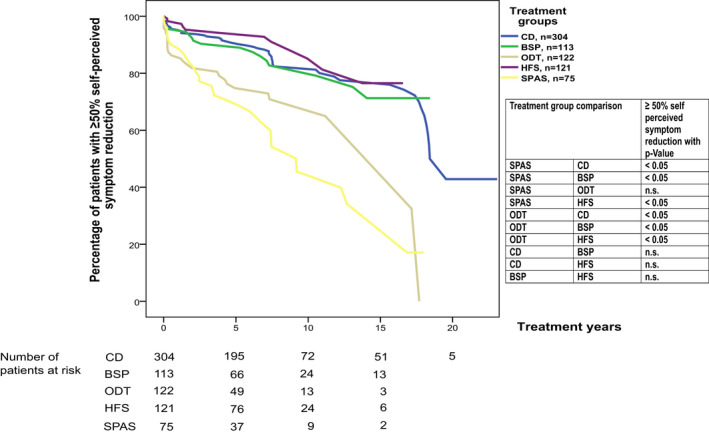
Kaplan–Meier curves displaying the cross‐sectional assessments by duration of prior therapy for the different indications (CD, BSP, ODT, HFS, and SPAS) show the probability of having ≥50% self‐perceived symptom reduction. Self‐perceived symptom reduction comparisons of the treatment groups over the years are presented. *P*‐values calculated by Cox proportional hazard models correcting for age at the first injection. Number of censored patients: CD 251, BSP 95, ODT 93; HFS 109, SPAS 44.

Global self‐perceived symptom reduction was not available for all patients; thus, this analysis was performed on a subset of patients: 304/527 (58%) CD, 113/173 (65%) BSP, 122/283 (43%) ODT, 121/184 (66%) HFS, and 75/184 (41%) SPAS patients could be included. The Kaplan–Meier analysis revealed that 74% of the patients with CD, 71% of patients with BSP, and 77% of patients with HFS experienced a stable reduction of self‐perceived symptoms of at least 50% with BoNT‐A treatment over 16 years of treatment. SPAS and ODT patients had significantly higher rates of insufficient treatment response, as reflected by indicating <50% self‐perceived symptom reduction in 83% of patients at 16 years (*P* < 0.05 Cox proportional hazard models correcting for age at the first injection), than BSP, HFS, and CD patients. SPAS patients showed a significantly earlier decline (*P* < 0.05, Cox proportional hazard models correcting for age at first injection) as at least 50% self‐perceived symptom reduction was already reached by 7 years of treatment in 60% of the patients compared to 16 years for CD, BSP, and HFS patients (Fig. 2).

### Global self‐perceived quality of life

**Figure 3:**
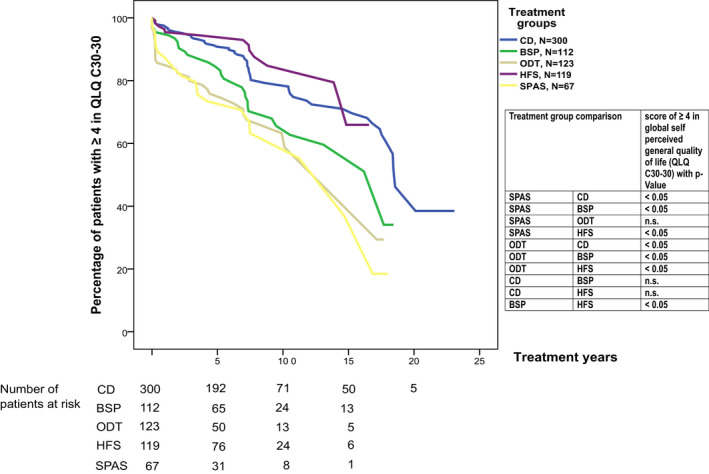
Kaplan–Meier curves displaying the cross‐sectional assessments by duration of prior therapy for the different indications (CD, BSP, ODT, HFS, and SPAS) show the probability of having at least a score of ≥4 in self‐perceived global general quality of life (QLQ C30 item 30). The self‐perceived global general quality of life comparisons of the treatment groups over the years are presented, *P*‐values calculated by Cox proportional hazard models correcting for age at the first injection. Number of censored patients: CD 241, BSP 81, ODT 91; HFS 105, SPAS 46.

To investigate the dynamics of patients’ global self‐perceived QoL, we investigated the probability of patients presenting at least medium (≥4) global self‐perceived general QoL (QLQ C30‐30). For this Kaplan–Meier analysis, 300/527 (57%) CD, 112/173 (65%) BSP, 123/283 (43%) ODT, 119/184 (65%) HFS, and 67/184 (36%) SPAS patients were available. The Kaplan–Meier curves indicated that SPAS and ODT patients experienced a significantly higher risk of dropping under a QoL (OLQ C30‐30) score of 4 (on a scale from 1 to 7) than CD and HFS patients. BSP patients had a significantly higher risk of decline in global QoL than HFS patients but significantly less decline in global QoL than ODT and SPAS patients (*P* < 0.05, Cox proportional hazard models correcting for age at the first injection). Sixty‐eight percent of the patients with CD and 66% of the patients with HFS experienced at least medium global QoL (≥4) after 16 years of treatment, whereas 40% of patients already reached a scale of <4 at 10 years for BSP, ODT, and SPAS (Fig. 3).

### Correlations of dosing and treatment outcomes

The self‐perceived symptom reduction assessed by patients was negatively correlated with the clinical global impression of severity evaluated by the treating physician in all indications, suggesting that the low severity assessed by the treater was associated with good subjective response to therapy: CD (Spearman: *r* ^2^ = −0.595, *P* < 0.001), BSP (*r* ^2^ = −0.621, *P* < 0.001), ODT (*r* ^2^ = −0.673, *P* < 0.001), HFS (*r* ^2^ = −0.554, *P* < 0.001), and SPAS (*r* ^2^ = −0.635, *P* < 0.001).

Furthermore, patients’ self‐perceived symptom reduction was moderately and positively correlated with global self‐perceived quality of health (QLQ C30‐29) in CD (*r* ^2^ = 0.346, *P* < 0.001), BSP (*r* ^2^ = 0.348, *P* < 0.001), ODT (*r* ^2^ = 0.417, *P* < 0.001), and SPAS (*r* ^2^ = 0.336, *P* < 0.001) as well as with global self‐perceived QoL (QLQ C30‐30) in CD (*r* ^2^ = 0.413, *P* < 0.001), BSP (*r* ^2^ = 0.449, *P* < 0.001), ODT (*r* ^2^ = 0.453, *P* < 0.001), and SPAS (*r* ^2^ = 0.462, *P* < 0.001).

**Figure 4:**
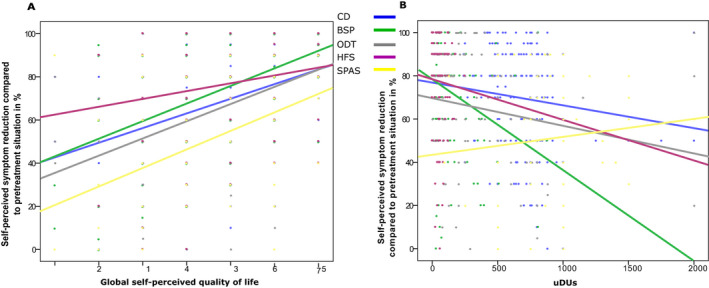
(A) Compound scatter plots with linear regression lines of self‐perceived symptom reduction assessed by the patient on the *y*‐axis and global self‐perceived quality of life (QLQ C30‐30) on the *x*‐axis in the different groups of disorders (CD, BSP, ODT, HFS, and SPAS). (B) Compound scatter plot with linear regression lines of the self‐perceived symptom reduction assessed by the patient on the y‐axis and unified dose units of the mean dose per session on the *x*‐axis in the different treatment groups (CD, BSP, ODT, HFS, and SPAS).

Exemplary scatter plots of self‐perceived symptom reduction assessed by the patient and global self‐perceived QoL (QLQ C30‐30) in the different groups of disorders are presented in Figure 4A. The plot shows that a high self‐perceived symptom reduction led to a high global self‐perceived QoL in all disease subgroups.

Correlation analysis of the unified dose units of the mean dose per session revealed negative correlations with self‐perceived symptom reduction assessed by the patients, indicating that patients with weaker self‐perceived response to therapy were treated with higher doses for all treatment groups (CD (*r* ^2^ = −0.233, *P* < 0.001), BSP (*r* ^2^ = −0.279, *P* < 0.001), ODT (*r* ^2^ = −0.233, *P* < 0.001), and HFS (*r* ^2^ = −0.240, *P* = 0.008), all Spearman, Fig. 4B) except for SPAS patients, who showed no correlation (*r* ^2^ = 0.033, *P* = 0.777). To detect factors influencing the mean single uDUs, a stepwise multilevel linear regression model analyzing treatment group, treatment adherence, global self‐perceived symptom reduction, clinical global impression of symptom severity, global self‐perceived quality of health, global self‐perceived QoL, treatment years, and MHDA‐AB status was performed.

Significant predictors for mean single uDUs were treatment indication (*P* < 0.001), MHDA‐AB status (*P* < 0.001), and global self‐perceived symptom reduction (*P* < 0.001) explaining 20% of the variance of the uDU values (*r* ^2^ = 0.200, corrected *r* ^2^ = 0.194; *F* (df 3, 411) = 34.212).

No additional significant influence was revealed for treatment adherence, clinical global impression of symptom severity, global self‐perceived quality of health, global self‐perceived QoL, and treatment years (*P* ≥ 0.05).

### Cross‐sectional comparisons between groups

An analysis of the clinical global impression of severity evaluated by the treating physician and the self‐perceived symptom reduction assessed by the patient revealed a mean of 25–50% global impression of severity under therapy and a mean of 50–80% self‐perceived symptom reduction, respectively (Table 1).

**Table 1:** Mean and standard deviation (SD) is presented for the parameters unified dose units (uDUs), clinical global impression of severity by the treating physician, and self‐perceived symptom reduction assessed by the patient of the different treatment groups. The results of the statistical analysis comparing these parameters between the different groups are presented in the lower half of the table.

Treatment groups	*n*	Mean uDUs	SD	*n*	Mean clinical global impression of severity evaluated by the treating physician in %	SD	Mean self‐perceived symptom reduction assessed by the patient in %	SD
CD	528	813.3	555.65	303	31.42	25	67.90	23.69
BSP	173	127.28	191.15	110	27.14	25.58	72.30	25.98
ODT	283	423.9	415.05	120	40.92	32.57	64.38	28.22
HFS	184	83.23	193.15	120	25.97	23.5	77.21	22.92
SPAS	184	938.6	370.93	74	47.57	29.44	51.62	29.14

Comparison of treatment groups		Mean uDUs	Mean clinical global impression of severity evaluated by the treating physician in %	Mean self‐perceived symptom reduction assessed by the patient in %
SPAS	CD	<0.05	<0.05	<0.05
SPAS	BSP	<0.05	<0.05	<0.05
SPAS	ODT	<0.05	n.s.	<0.05
SPAS	HFS	<0.05	<0.05	<0.05
ODT	CD	<0.05	n.s.	n.s.
ODT	BSP	<0.05	<0.05	n.s.
ODT	HFS	<0.05	<0.05	<0.05
CD	BSP	<0.05	n.s.	n.s.
CD	HFS	<0.05	n.s.	<0.05
BSP	HFS	<0.05	n.s.	n.s.

*P*‐values for group comparisons (ANCOVA with Bonferroni confidence interval adjustment and correction for age at the first injection and treatment duration).

SPAS and ODT patients received the most severe ratings of clinical global impression of severity by the treating physician and had the lowest self‐perceived symptom reduction. The differences were significant between SPAS and HFS/CD/BSP patients as well as between ODT and HFS patients (Table 1).

### Patients no longer on follow‐up

A total of 671 patients were no longer on follow‐up at our outpatient clinic (defined as ≥6 months without visits) at the time of cross‐sectional assessments of global self‐perceived general QoL and global self‐perceived symptom reduction. We attempted to contact all of these patients by telephone, and 143 (21.3%) patients could be reached and gave consent to a telephone interview regarding their current treatment. These patients reported receiving no specific treatment (35%), BoNT therapy at another clinic (27.3%), alternative non‐neurological treatment (e.g., alternative medicine or physiotherapy) (16.8%), oral medication (e.g., anticholinergics or antispasticity drugs) (8.4%), or surgical treatment (4.9%), or they eventually returned to our clinic (7.7%) (Table 2).

**Table 2:** Analysis of all patients and the treatment groups no longer on follow‐up at our outpatient clinic, including the number of successfully contacted patients and their current treatment after leaving our outpatient clinic.

	All treatment groups	CD	BSP	ODT	HFS	SPAS
Patients no longer on follow‐up at our outpatient clinic (% of analyzed treatment group patients)	671 (50%)	174 (33.5%)	58 (36%)	177 (62.5%)	77 (41.8%)	117 (63.6%)
No. of successfully contacted patients (% of patients no longer on follow‐up)	143 (21.3%)	51 (22.7%)	14 (24.1%)	35 (19.8%)	21 (27.3%)	22 (18.8%)
No specific treatment patients (% of successfully contacted patients no longer on follow‐up)	50 (35.0%)	12 (23.5%)	7 (50%)	17 (48.6%)	9 (42.9%)	5 (22.7%)
BoNT treatment outside our BoNT clinic (% of successfully contacted patients no longer on follow‐up)	39 (27.3%)	17 (33.3%)	7 (50%)	5 (14.3%)	6 (28.6%)	4 (18.2%)
Deep brain stimulation or surgery (% of successfully contacted patients no longer on follow‐up)	7 (4.9%)	5 (9.8%)	0 (0%)	1 (2.9%)	1 (4.8%), microvascular decompression	0 (0%)
Oral medication (% of successfully contacted patients no longer on follow‐up)	12 (8.4%)	7 (13.7%)	0 (0%)	4 (11.4%)	0 (0%)	1 (4.5%)
Alternative non‐neurological treatment (% of successfully contacted patients no longer on follow‐up)	24 (16.8%)	7 (13.7%)	0 (0%)	5 (14.3%)	2 (9.5%)	10 (45.5%)
Return to our BoNT clinic (% of successfully contacted patients no longer on follow‐up)	11 (7.7%)	3 (5.9%)	0 (0%)	3 (8.6%)	3 (14.3%)	2 (9.1%)

### Effect of MHDA‐ABs against BoNT‐A

**Figure 5:**
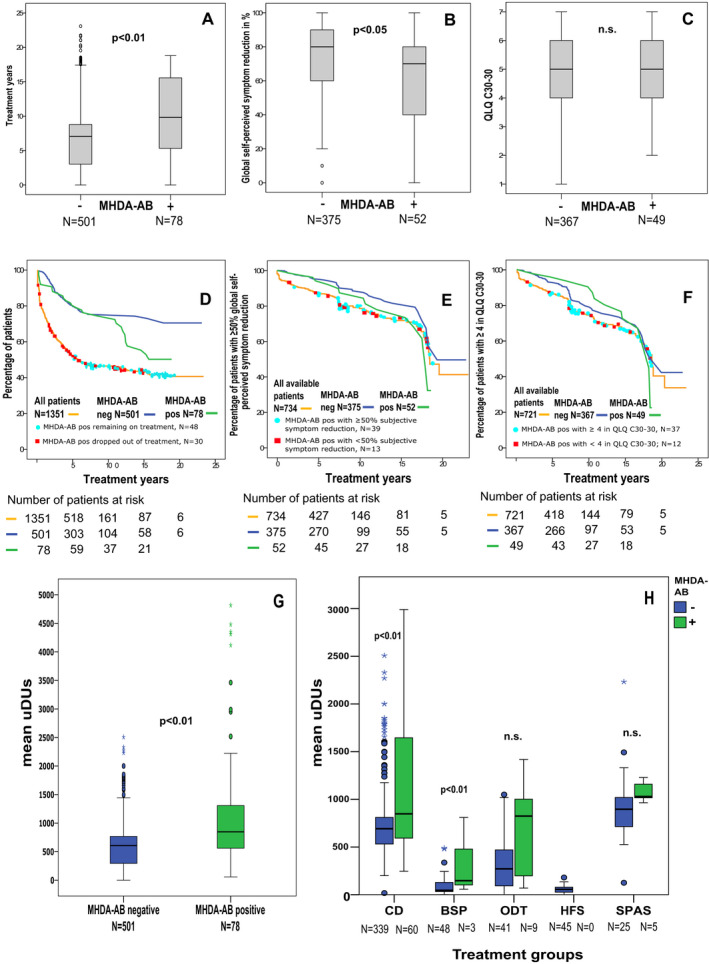
Comparison of MHDA‐AB‐positive and MHDA‐AB‐negative patients. For all boxplots, the horizontal lines in the middle of the boxplots demonstrate the medians of the patients with and without MHDA‐ABs. The IQR is presented by the box, and the minimum and maximum values are presented by whiskers (excluding outliers). Outliers, defined as values 1.5 to 3.0 times outside the IQR, are presented as circles, and extreme outliers, defined as values of more than 3.0 times outside the IQR, are presented as asterisks. (A) MHDA‐AB‐positive patients had been treated with BoNT‐A significantly longer than MHDA‐AB‐negative patients (Mann–Whitney *U* test with Bonferroni correction for multiple group comparisons). (B) MHDA‐AB‐positive patients showed significantly less global self‐perceived symptom reduction (0–100%) than MHDA‐AB‐negative patients (Mann–Whitney *U* test with Bonferroni correction for multiple group comparisons). (C) No difference in global self‐perceived quality of life (QLQ C30‐30 ranging from 1 to 7) was detected between MHDA‐AB‐positive and MHDA‐AB‐negative patients. (D) The orange Kaplan–Meier curve shows the probability of all patients remaining on treatment at follow‐up. MHDA‐AB‐positive patients are indicated by colored dots placed at the time point of the last follow‐up; blue dots indicate patients continuing BoNT treatment, and red rectangles indicate treatment dropouts. More than half of the MHDA‐AB‐positive patients remained on therapy despite positivity. Number of censored patients: 699. The blue Kaplan–Meier curve represents the probability of MHDA‐AB‐negative patients remaining on treatment (number of negative‐MHDA‐AB patients censored: 390), whereas the green Kaplan–Meier curve demonstrates the probability of MHDA‐AB‐positive patients remaining on therapy (number of MHDA‐AB‐positive patients censored: 48). (E) The orange Kaplan–Meier curve shows the probability of all available patients having ≥50% self‐perceived symptom reduction. The results of MHDA‐AB testing at the last follow‐up are presented with colored dots placed at the time point of the last visit; blue dots indicate patients still with ≥50% self‐perceived symptom reduction, and red rectangles indicate patients with <50% self‐perceived symptom reduction. Number of censored patients: 591. The blue Kaplan–Meier curve represents the probability of MHDA‐AB‐negative patients having ≥50% self‐perceived symptom reduction (number of negative‐MHDA‐AB patients censored: 330), whereas the green Kaplan–Meier curve demonstrates the probability of MHDA‐AB‐positive patients having ≥50% self‐perceived symptom reduction (number of MHDA‐AB‐positive patients censored: 37). (F) The orange Kaplan–Meier curve shows the probability of all available patients having ≥4 in self‐perceived global QoL (QLQ C30‐30). The results of MHDA‐AB testing at the last follow‐up are presented with colored dots placed at the time point of the last visit; blue dots indicate patients still with ≥4 self‐perceived global QoL (QLQ C30‐30), and red rectangles indicate patients with <4 self‐perceived global QoL (QLQ C30‐30). Number of censored patients: 564. The blue Kaplan–Meier curve represents the probability of MHDA‐AB‐negative patients having ≥4 in self‐perceived global QoL (QLQ C30‐30) (number of MHDA‐AB‐negative patients censored: 290), whereas the green Kaplan–Meier curve demonstrates the probability of MHDA‐AB‐positive patients having ≥4 in self‐perceived global QoL (QLQ C30‐30) (number of MHDA‐AB‐positive patients censored: 37). (G) Overall mean unified dose units (uDUs) per treatment session in all MHDA‐AB‐tested patients (negative, blue: positive, and green) were analyzed. The analysis revealed significantly higher mean uDUs in MHDA‐AB‐positive patients than in MHDA‐AB‐negative patients. ANCOVA with Bonferroni confidence interval adjustment correcting for age at the first treatment and treatment duration. *P*‐values < 0.05 were considered statistically significant. (H) Mean uDUs per treatment session were analyzed for patients with the different neurological disorders and negativity (blue) and positivity (green) for MHDA‐ABs. The analysis revealed significantly higher mean uDUs in MHDA‐AB‐positive CD patients than in MHDA‐AB‐negative CD patients, as well as in MHDA‐AB‐positive BSP patients compared to MHDA‐AB‐negative BSP patients. In the other neurological diseases, no difference in mean uDUs was detected between MHDA‐AB‐positive and MHDA‐AB‐negative patients. ANCOVA with Bonferroni confidence interval adjustment correcting for age at first treatment and treatment duration. *P*‐values <0.05 were considered statistically significant.

We recently reported a high rate of MHDA‐ABs against BoNT‐A in long‐term treated patients,^14^ who were a subset (596, 44%) of this larger cohort (1351 patients). To investigate the effect of MHDA‐ABs on treatment adherence and treatment response, we compared treatment years, QLQ C30‐29, QLQ C30‐30, clinical global impression of severity judged by the treating physician, and global self‐perceived symptom reduction between MHDA‐AB‐negative and MHDA‐AB‐positive patients (for whom these data were available). An analysis of the different neurological diseases revealed significantly longer treatment years (Fig. 5A) in MHDA‐AB‐positive (9.99 ± 5.88 SD years) patients than in MHDA‐AB‐negative patients (7.16 ± 5.07 SD years) (*P* < 0.05 Mann–Whitney *U* with Bonferroni post hoc test). We observed a higher clinical global impression of symptom severity and less self‐perceived symptom reduction (Fig. 5B) for MHDA‐AB‐positive patients than for MHDA‐AB‐negative patients (*P* < 0.05 Mann–Whitney *U* with Bonferroni post hoc test). Global self‐perceived quality of health (QLQ C30‐29) and global self‐perceived QoL (QLQ C30‐30) (Fig. 5C) showed no difference between MHDA‐AB‐positive and MHDA‐AB‐negative patients.

Independent analysis of the different neurological disorders revealed a significant difference (*P* < 0.05 Mann–Whitney *U* with Bonferroni post hoc test) between MHDA‐AB‐positive and MHDA‐AB‐negative patients only for the global self‐perceived quality of health of ODT and for global self‐perceived symptom reduction in CD, whereas all other items showed no significant differences between MHDA‐AB‐positive and MHDA‐AB‐negative patients in all subgroups.

To analyze the relevance of MHDA‐ABs for treatment adherence and response to therapy over time, we plotted patients with prior positive MHDA‐AB testing on Kaplan–Meier curves for the probability of remaining on therapy (Fig. 5D), having ≥50% self‐perceived symptom reduction (Fig. 5E) and having ≥4 in self‐perceived global general QoL (QLQ C30‐30, Fig. 5F). Of the 579 patients with available data on MHDA‐AB status and treatment duration, we observed no differences in adherence treatment between the 78 MHDA‐AB‐positive and 501 MHDA‐AB‐negative patients for patients with <10 years of therapy; however, for patients with >10 years of therapy, MHDA‐AB‐positive patients presented a higher probability of dropping out of therapy (Fig. 5D). Plotting the MHDA‐AB‐positive patients on the curve for adherence to therapy of all patients (Fig. 5D) revealed that 30 (39%) MHDA‐AB‐positive patients dropped out of our BoNT treatment, while 47 (61%) of them remained on BoNT treatment despite being positive for MHDA‐ABs. Furthermore, patients with MHDA‐AB positivity within the first 5 years of therapy all dropped out of therapy, whereas most patients positive for MHDA‐ABs after 7 years of therapy remained on treatment despite the antibodies.

In contrast, the curves for the probability of having ≥50% self‐perceived symptom reduction (Fig. 5E) and having ≥4 in self‐perceived global general QoL demonstrated no significant differences between MHDA‐AB‐positive and MHDA‐AB‐negative patients and a more even distribution of MHDA‐AB‐positive patients, with over 50% of patients remaining at scores ≥50% or ≥4 despite being positive for MHDA‐ABs.

In a further analysis, the mean BoNT‐A uDU per treatment session of MHDA‐AB‐positive and MHDA‐AB‐negative patients was investigated for the different neurological disorders. MHDA‐AB‐positive patients were treated with significantly higher uDUs than MHDA‐AB‐negative patients (*P* < 0.01, ANCOVA with Bonferroni confidence interval adjustment correcting for age at the first treatment and treatment duration) in the analysis of all patients (78 MHDA‐AB‐positive patients with mean uDUs 1203.28 ± 1072.50 SD vs. 501 MHDA‐AB‐negative patients with mean uDUs 595.75 ± 415.15). Independent analysis of the patients by treatment indication revealed that significant differences between MHDA‐AB‐positive and MHDA‐AB‐negative patients were observed only for the CD subgroup (60 MHDA‐AB‐positive patients with mean uDUs 1348.93 ± 1161.93 SD vs. 339 MHDA‐AB‐negative patients with mean uDUs 744.18 ± 342.2 SD) and for the BSP subgroup (3 MHDA‐AB‐positive patients with mean uDUs 338.18 ± 411.13 SD vs. 48 MHDA‐AB‐negative patients with mean uDUs 92.58 ± 109.28 SD), whereas no significant differences were detected for the other treatment groups (Fig. 5G and H).

In a power analysis for an actual power of 0.95 (two‐tailed 1‐*β*), the estimated sample size of MHDA‐AB‐positive and MHDA‐AB‐negative patients in the comparison of uDUs was 96 patients (48 for each group) for all patients, 108 patients (54 for each group) for CD patients, 80 (40 for each group) for BSP patients, 72 (36 for each group) for ODT patients, and 170 (85 for each group) for SPAS patients, suggesting that our analysis was underpowered for the analysis of the subgroups except for CD and ODT.

## Discussion

To the best of our knowledge, the present study is the largest monocentric study analyzing longitudinal real‐world data on long‐term BoNT‐A treatment dosing and therapy adherence. We included 1351 patients with up to 23 years of follow‐up covering five different neurological diseases. Furthermore, we provided cross‐sectional data on global self‐perceived QoL and symptom reduction. A strength of our study was the large sample size, the monocentric design assuring the homogeneity of data, and the long treatment duration, with several of our patients being treated for over 20 years. This is of particular importance, as comparative data on long‐term adherence to therapy and PROs covering different indications are extremely rare.^1^, ^7^, ^8^, ^9^

Our analysis of long‐term adherence to BoNT‐A therapy revealed that most treatment dropouts occurred within the first 8 years of therapy for all indications. Long‐term therapy adherence was better in BSP, HFS, and CD patients than in ODT and SPAS patients. Fifty‐nine percent of the 173 BSP and 52% of the 184 HFS patients remained on treatment for over 16 years, and 45% of the 527 CD patients remained on treatment for over 23 years, whereas only 25% of the 283 ODT and 28% of the 184 SPAS patients remained on therapy for over 16 years.

Possible general reasons for these observations included patients losing interest in a therapy that did not sufficiently relieve their symptoms; the achievement of therapy goals; incapability to return to the clinic owing to organizational issues (e.g., transportation, especially for more disabling disorders such as SPAS); shorter therapy duration due to older age, earlier death or progression of disability (e.g., by further strokes, or other comorbidities, especially in SPAS); or a combination of all of the above reasons.

In addition, the self‐perceived symptom reduction by long‐term BoNT‐A treatment over the years appeared to last longer in BSP, HFS, and CD patients than in patients with SPAS and ODT, suggesting that self‐perceived efficacy of therapy may play a role in the adherence of our patients.

As higher mean single doses per session of BoNT‐A were used in SPAS patients than in the patients with other indications, the risk of developing MHDA‐ABs against BoNT‐A was higher, as recently reported,^14^ possibly resulting in a higher risk of secondary treatment failure and dropping out of therapy. It is important to mention that, in contrast to previous reports,^12^, ^17^, ^18^ the MHDA‐AB‐positive patients in our cohort did not completely lose their response to BoNT therapy. In fact, more than two‐third of the MHDA‐AB‐positive patients still experienced ≥50% self‐perceived symptom reduction. However, in the overall analysis, MHDA‐AB‐positive patients had significantly less self‐perceived symptom reduction than MHDA‐AB‐negative patients, suggesting that MHDA‐ABs did indeed have an impact on the response to therapy, in line with previous reports.^12^, ^17^, ^18^ A strength of our study is that binding antibody screening was performed by ELISA, but only neutralizing antibodies as confirmed by the MHDA were included for analysis. However, the MHDA is more sensitive than other antibody tests such as the mouse protection assay, and therefore MHDA‐AB‐positive patients regularly show at least a moderate response to therapy as evidenced by our data. When the patients were analyzed separately by subgroups, these differences between MHDA‐AB‐positive and MHDA‐AB‐negative patients lost significance, presumably due to the lower sample sizes (a G*Power 3 analysis revealed that all of our subanalyses were underpowered). For the overall cohort and the CD subgroup, MHDA‐AB‐positive patients were treated with significantly higher mean single doses per session than MHDA‐AB‐negative patients, suggesting that the effect of MHDA‐ABs can possibly be compensated by increasing the doses of BoNT‐A.

Moreover, higher doses of BoNT‐A could have increased the risk of developing MHDA‐ABs in the first place. The differences in BoNT‐A dosing between MHDA‐AB‐positive and MHDA‐AB‐negative patients were not significant for the other treatment groups, again probably because of the low numbers of MHDA‐AB‐positive patients in these groups, which again is supported by the power analysis. We acknowledge that the results of the subgroup analysis of the effects of MHDA‐ABs in the treatment groups except for the CD group should be interpreted with great caution due to the low numbers of MHDA‐AB‐positive patients in these groups.

We concluded that MHDA‐ABs had a significant impact on global self‐perceived symptom reduction, but with only a limited degree.

Along with the course of therapy, an unsatisfactory treatment response, also in the absence of MHDA‐ABs, is often counteracted by increasing the doses to be injected. In line with this, our analysis revealed a weak but significant correlation between dose and self‐perceived symptom reduction, with higher mean single uDUs being associated with worse self‐perceived symptom reduction for all indications except for SPAS, for which no correlation was detected. The factors significantly influencing the uDUs injected were treatment indication, MHDA‐AB status, and global self‐perceived symptom reduction. Prospective studies on larger cohorts are warranted to address this issue.

Another major factor influencing the response to therapy was the complexity of the disorder and of the required injection patterns. In our cohort, the ODT group received lower BoNT‐A doses per session than our CD patients but had comparatively lower adherence to therapy and less self‐perceived symptom reduction over time. The reason may be that the ODT subgroup was composed of a more heterogeneous group of dystonias. Possibly, the diversity of this ODT group and the complexity of the required injection patterns made it harder to maintain good and stable self‐perceived symptom reduction over time, which then resulted in lower adherence to therapy. These reasons may also apply to our SPAS patients, who also often present complex symptoms and injection patterns.

Considering the treatment duration of some patients and a consistent mean QLQ C30‐30 score of ≥4 throughout all therapeutic groups and intervals, long‐term usage of BoNT‐A appeared to at least maintain QoL, which was consistent with previously published studies on the long‐term efficacy of BoNT‐A treatment.^8^, ^9^, ^19^, ^20^

The EORTC global self‐perceived health status (QLQ C30‐29) and global self‐perceived QoL (QLQ C30‐30) were selected because they can be assessed very rapidly and are not disease‐specific, which, on the one hand, might be an advantage for the study of patients with heterogeneous disorders who receive BoNT‐A treatment for different indications. However, the meaningfulness was limited because we investigated only two questions of the EORTC QLQ C30. The limited specificity of these measures may also be associated with sensitivity reduction for the unique challenges of the different indications, particularly when these indications are grouped.^21^ A further limitation is that no clinical assessment of the patient by the physician was performed at the time point of the maximum treatment effect. Furthermore, the period of assessment differed between self‐perceived symptom reduction by the patients, which was focused on the period since the last BoNT injection, and the patients’ symptoms assessed by the treating physicians, which was performed at the appointment for BoNT reinjection.

To reduce selection bias when analyzing only patients on therapy, we chose to also include patients who had previously dropped out of follow‐up at our center. The majority of patients who dropped out received no specific further treatment, BoNT treatment outside of our outpatient clinic, or non‐neurological treatment options.

However, we have to acknowledge that the percentage of patients available for telephone interviews (143 of 671, 21.3%) was rather low. This was most likely due to the often long time lapse between dropping out of therapy and the time of our assessments.

Another limitation of our study was the retrospective design and the lack of a control group, which made it impossible to exclude confounding factors such as aging, coexisting comorbidities, additional medications, and/or procedures, which might have influenced global QoL. As an example, health utility naturally declines with age within the general population.^22^, ^23^

Furthermore, we acknowledge that the conversion calculation of uDUs by multiplying the inco‐BoNT and ona‐BoNT units by 2.5 to reach comparability with abo‐BoNT is heterogeneously performed and controversially discussed in the literature. However, our conversion rate was in line with that of several previous studies.^14^, ^15^, ^16^

This study investigated a large variety of neurological disorders with different disease severity, progression, and variable treatment histories as well as dosing regimens. These and the abovementioned factors added confounding variability to the interpretation of our data. However, despite these limitations, they also contributed to the strengths of the study, as PROs of real‐world clinical practice with all its complexity were analyzed and reported.

In summary, our study retrospectively examined the long‐term results of therapy adherence, global QoL, and self‐perceived symptom reduction after BoNT treatment involving a wide range of indications, allowing comparative analyses. SPAS and ODT patients presented fewer positive outcomes compared to HFS, BSP, and CD patients. MHDA‐ABs against BoNT had only limited effects on the outcomes assessed. Our data indicate that in a relevant proportion of patients with neurological disorders, BoNT therapy can help to improve and maintain self‐perceived symptom reduction and QoL over substantial periods of time.

## Author Contributions

JL, AJ, HH, and PA designed the study, acquired and analyzed the data, created the figures, conducted literature research, and wrote the manuscript. JL, GS, and PA performed the statistical analysis. All authors (JL, AJ, SS, UK, MM, MR, GS, HB, OA, AM, JW, SJ, MG, AS, HPH, HH, and PA) drafted a significant portion of the manuscript or figures, interpreted the data, critically revised the manuscript for important intellectual content, and approved the final version of the manuscript.

## Conflict of Interests

John‐Ih Lee has received honoraria for speaking/consultation from Bayer Healthcare, Boehringer Ingelheim, Allergan, Novartis, Ipsen, Teva, and Daiichi‐Sankyo as well as travel grants from Bayer Healthcare, Merz, Allergan, and Ipsen outside the submitted work. Allergan, Ipsen, and Merz manufacture the drugs that were used in this study. Alexander Jansen declares no disclosures relevant to the manuscript. Sara Samadzadeh declares no disclosures relevant to the manuscript. Ulrike Kahlen has received travel grants from Merz, Allergan, and Ipsen outside the submitted work. These companies manufacture the drugs that were used in this study. Marek Moll has received travel grants from Merz outside the submitted work. This company manufactures one of the drugs that were used in this study. Marius Ringelstein received speaker honoraria from Novartis, Bayer Vital GmbH, Roche, Alexion, and Ipsen and travel reimbursement from Bayer Schering, Biogen Idec, Merz, Genzyme, Teva, Roche, and Merck outside the submitted work. Ipsen and Merz manufacture drugs that were used in this study. Giulia Soncin declares no disclosures relevant to the manuscript. Hans Bigalke works for Toxogen and declares grant, personal fee, and nonfinancial support from Allergan, Ipsen, and Merz Pharmaceuticals outside the submitted work. These companies manufacture the drugs that were used in this study. Orhan Aktas has received honoraria for speaking/consultation and travel grants from Bayer Healthcare, Biogen Idec, Chugai, Novartis, Medimmune, Merck Serono, and Teva and research grants from Bayer Healthcare, Biogen Idec, Novartis, and Teva outside the submitted work. Alexia‐Sabine Moldovan has received travel grants from Merz, Allergan, and Ipsen outside the submitted work. These companies manufacture the drugs that were used in this study. Julia Waskoenig has received travel grants from Allergan and Ipsen outside the submitted work. These companies manufacture the drugs that were used in this study. Sebastian Jander has received honoraria for speaking/consultation from Bayer Healthcare, Boehringer Ingelheim, Bristol‐Myers Squibb, Pfizer, Biogen, Alexion, and Daiichi‐Sankyo as well as travel grants from Bayer Healthcare and Daiichi‐Sankyo outside the submitted work. Michael Gliem has received honoraria for speaking/consultation from Bayer Healthcare and Boehringer Ingelheim and a research grant from B. Braun outside the submitted work. Alfons Schnitzler has received honoraria for speaking/consulting from Medtronic, Boston Scientific, Abbott/SJM, Grünenthal, AbbVie, UCB, MEDA Pharma, GlaxoSmithKline, and Teva. Hans‐Peter Hartung has, outside the work presented, received fees for serving on steering or data monitoring committees from Bayer Healthcare, Biogen, Celgene Receptos, GeNeuro, Sanofi Genzyme, Merck, Novartis, Octapharma, Teva Pharmaceuticals, MedImmune, and Roche; fees for serving on advisory boards from Biogen Idec, Sanofi Genzyme, Merck, Novartis Pharmaceuticals, Octapharma, Teva Pharmaceuticals, and Roche; and lecture fees from Biogen, Sanofi Genzyme, Merck, Novartis Pharmaceuticals, Octapharma, Teva Pharmaceuticals, MedImmune, and Roche. Harald Hefter has been supported by a restricted grant of the Inge‐Diesbach‐Stiftung. This grant did not influence the design and content of the present study. He declares no other disclosures relevant to the manuscript. Philipp Albrecht reports grants from Ipsen, grants from Merz Pharmaceuticals, for MHDA‐AB testing and analysis of the data of patients with spasticity, respectively, during the conduct of the study; grants and personal fees from Allergan, grants and personal fees from Biogen, grants and personal fees from Merck, grants and personal fees from Novartis, grants and personal fees from Roche, personal fees from Teva, grants, personal fees, and nonfinancial support from Celgene, personal fees from Bayer Healthcare, grants and personal fees from Sanofi Aventis Genzyme, outside the submitted work; Allergan, Ipsen, and Merz manufacture the drugs that were used in this study.

## Acknowledgement

Open access funding enabled and organized by Projekt DEAL.
